# The Complexity of Sporadic Alzheimer's Disease Pathogenesis: The Role of RAGE as Therapeutic Target to Promote Neuroprotection by Inhibiting Neurovascular Dysfunction

**DOI:** 10.1155/2012/734956

**Published:** 2012-03-11

**Authors:** Lorena Perrone, Oualid Sbai, Peter P. Nawroth, Angelika Bierhaus

**Affiliations:** ^1^Laboratoire des Neurobiologie des Interactions Cellulaires et Neurophysiopathologie (NICN), CNRS, UMR6184, Boulevard Pierre Dramard, 13344 Marseille, France; ^2^Department of Medicine 1, University Hospital of Heidelberg, 69120 Heidelberg, Germany

## Abstract

Alzheimer's disease (AD) is the most common cause of dementia. Amyloid plaques and neurofibrillary tangles are prominent pathological features of AD. Aging and age-dependent oxidative stress are the major nongenetic risk factors for AD. The beta-amyloid peptide (A*β*), the major component of plaques, and advanced glycation end products (AGEs) are key activators of plaque-associated cellular dysfunction. A*β* and AGEs bind to the receptor for AGEs (RAGE), which transmits the signal from RAGE via redox-sensitive pathways to nuclear factor kappa-B (NF-*κ*B). RAGE-mediated signaling is an important contributor to neurodegeneration in AD. We will summarize the current knowledge and ongoing studies on RAGE function in AD. We will also present evidence for a novel pathway induced by RAGE in AD, which leads to the expression of thioredoxin interacting protein (TXNIP), providing further evidence that pharmacological inhibition of RAGE will promote neuroprotection by blocking neurovascular dysfunction in AD.

## 1. Introduction

Alzheimer's disease (AD) pathology is characterized in by the presence of several kinds of amyloid plaques and neurofibrillary tangles in the brain, which are mainly composed by the beta amyloid (A*β*), derived from the proteolytic cleavage of the amyloid precursor protein (APP), and hyperphosphorylated tau [1]. AD can be subdivided in 2 major forms: (i) familial AD, which represents rare early onset forms due to gene mutations leading to enhanced A*β* production or faster aggregating A*β* peptide; (ii) sporadic AD forms, which represent about 95% of AD cases [2]. The pathogenesis of sporadic AD is extremely complex, and its ultimate cause is still under debate. Epidemiological studies reveal growing evidence that most cases of sporadic AD likely involve a combination of genetic and environmental risk factors. However, the only risk factors so far validated for late-onset disease are age, family history, and the susceptibility gene ApoE4 allele [3].

A hallmark of the aged brain is the presence of oxidative stress [4]. A*β* fibrils are toxic by generating oxygen free radicals in the absence of any cellular element [5, 6]. However, synaptic dysfunction and behavioral changes in AD precede the formation of large A*β* aggregates and fibrils. Indeed, A*β* dimers and soluble oligomers are considered the major toxic form [7, 8], while fibrils-induced oxidative stress operates late in the course of AD. Thus, the mechanisms through which A*β* exerts its toxic effect at the early stages of AD remain still to be clarified. Recent evidences suggest that age-relate cofactors play a key function in mediating the toxicity of A*β* at early, AD stages. One of the risk factors is diabetes mellitus (DM) and several studies demonstrated a link between DM and AD [9–11]. In agreement, both hyperglycemia in DM and age-dependent oxidative stress induce the formation of advanced glycation end products (AGEs) [12, 13]. AGEs derive from a multistep reaction of reducing sugars or dicarbonyl compounds with the amino groups of proteins [13]. AGEs accumulate in AD brain and accelerate A*β* deposition [14, 15]. It has been shown that the interaction of AGEs with their receptor (RAGE) induces the production of reactive oxygen species (ROS), participating to the early toxic events that lead to AD progression [16]. RAGE is a multiligand receptor of the immunoglobulin superfamily of cell surface molecules acting as counterreceptor for various ligands, such as AGEs, S100/calgranulins, HMGB1 proteins, A*β* peptides, and the family of beta-sheet fibrils [17, 18]. Its ectodomain is constituted by one V-type followed by two C-type domains. The N-terminal V-domain seems to be implicated in the recognition of RAGE ligands [19]. Studies with RAGE−/− mice confirmed that RAGE contributes to AD [20, 21]. Notably, diabetic AD patients show enhanced cell damage, which is RAGE dependent [11]. Thus, RAGE seems to represent an excellent cofactor promoting A*β*-induced cellular dysfunction.

Several studies indicate that RAGE induces neurodegeneration in AD via multiple pathways. In AD brain, RAGE is evident in neurons, microglia, astrocytes, and in brain endothelial cells [19, 22]. The activation of RAGE expressed in neuronal cells promotes synaptic dysfunction. RAGE also promotes neurodegeneration by inducing inflammation in glial cells. Moreover, RAGE is responsible of the transport of A*β* from the blood to the brain [23], inducing cerebrovascular dysfunction that ultimately results in neurovascular inflammation and subsequent synaptotoxicity [24]. Notably, the G82S RAGE allele (a polymorphism in RAGE sequence) is associated with increased risk of AD [25], supporting the hypothesis that RAGE is implicated in the progression of sporadic AD. At early stages of AD, when the level of A*β* and AGEs are low, RAGE amplifies their effects on different cell types, ultimately contributing to neuronal dysfunction and neurodegeneration. Different animal models have been analyzed to decipher the role of RAGE in AD progression: (i) injection of AGEs into the rat hippocampus; (ii) injection of A*β* in rat hippocampus; (iii) various transgenic (Tg) mice expressing one or more gene variant of the amyloid precursor protein (APP); (iv) presenilins, which are implicated in APP cleavage and A*β* production leading to amyloid plaque formation; (v) tau that forms the characteristic tangles when is hyperphosphorylated. In addition, the brain of animal model of diabetes was analyzed to find the link between DM and AD.

We recently demonstrated that RAGE triggering induces the expression of thioredoxin interacting protein (TXNIP) in various cell types, promoting inflammation [26, 27]. TXNIP binds to thioredoxin (TRX) and inhibits its anti-oxidant activity, leading to oxidative stress in various cell type [28]. We demonstrated that oxidative stress plays a key function in AD progression [6, 29]. TXNIP expression is enhanced in several disease risk for AD: diabetes [26, 28, 30], hypertension [31], and ischemia [32]. Insulin is necessary to maintain normal brain function, and peripheral insulin resistance enhances the risk to develop AD, by affecting brain glucose metabolism, neurotransmitters levels, enhancing inflammation [33]. Interestingly, TXNIP is necessary to mediate insulin resistance in diabetes [34]. TXNIP is early overexpressed in the hippocampus of an AD mice model. Moreover, A*β* induces the RAGE-dependent expression of TXNIP in an in vitro model of the blood brain barrier (BBB).

 Notably, TXNIP and RAGE, both may exacerbate injury and inflammation when chronically activated, while they mediate neuronal repair when transiently expressed [26, 27]. Moreover, RAGE can also promote neurite outgrowth [35]. Thus, inhibition of chronic activation of RAGE and TXNIP can efficiently provide neuroprotection in AD.

## 2. Role of RAGE in Amplifying Age-Dependent Oxidative Stress

 Human aging is an inexorable biological phenomenon characterized by a progressive decrease in physiological capacity, and the reduced ability to respond to environmental stresses leads to increased susceptibility to disease. In 1956, Harman developed the free radical theory of aging [36] that argues that aging results from the damage generated by reactive oxygen species (ROS) [37]. According to this theory, aging is the result of accumulation of oxidative-damaged macromolecules (lipid, protein, DNA) due to the aerobic metabolism, which accumulate throughout lifetime [38]. Thus, aging is associated with imbalance between the rate of antioxidant defenses and intracellular concentration of ROS. The relevance role of ROS in aging consists in their ability to attack vital cell components like polyunsaturated fatty acids, proteins, and nucleic acids. These reactions can alter intrinsic membrane properties like fluidity, ion transport, loss of enzyme activity, protein cross-linking, and inhibition of protein synthesis, DNA damage, ultimately resulting in cell death. Many disorders, like cardiovascular diseases, rheumatoid arthritis, cancer, atherosclerosis, and AIDS, have been reported as the ROS-mediated disorders.

ROS has been also implicated in neurodegenerative diseases like Parkinson and Alzheimer diseases (AD). Indeed, the brain is particularly vulnerable to oxidative damage because of its high utilization of oxygen, increased levels of polyunsaturated fatty acid, and relatively high levels of redox transition metal ions; in addition, the brain has relatively low levels of antioxidants [39]. The presence of iron ion in an oxygen-rich environment can further lead to enhanced production of hydroxyl free radicals and ultimately lead to a cascade of oxidative events [6]. In the AD brain, the role of ROS has been well documented with markers for protein, DNA, RNA oxidation, and lipid peroxidation. In fact, increased reactive carbonyls were the first form of oxidative damage identified in AD [40]. Several studies showed the presence of additional protein markers like protein nitration supporting that nitrosative stress also contributes to neurodegeneration disease [39]. Amplified lipid peroxidation has been also described in several neurodegenerative diseases [41]. AD brains show an increase in free 4-hydroxy-2-trans-nonenal (HNE) in amygdala, parahippocampal gyrus, and hippocampus of the AD brain compared with age-matched controls [42]. In addition, DNA is a target of ROS, which leads to cellular aging. Oxidative damage to DNA induces strand breaks DNA-DNA and DNA-protein cross-linking and translocation. DNA bases are also attacked by the lipid peroxidation. This modification can cause inappropriate base leading to alter protein synthesis [43]. AGEs are considered important markers of oxidative stress and accumulating during aging and diseases, markers of carbonyl stress, which accumulate due to an increased level of sugars and reactive dicarbonyl compounds such as glucose, fructose, deoxyglucose, glyoxal, methylglyoxal, and triosephosphates [38, 44]. AGE formation begins when amino groups of proteins particularly the N-terminal amino group and side chains of lysine and arginine react nonenzymatically with these reactive carbonyl compounds [45]. This posttranslational modification, termed “non-enzymatic glycation” or “glycation,” derives from reversible Schiff-base adducts to protein through oxidations and dehydrations bound Amadori products. The irreversible formation of AGEs results in protease-resistant cross-linking of peptides, proteins, and other macromolecules. AGEs are localized in pyramidal neurons that appear to selectively accumulate AGEs in an age-dependant manner. In the AD brain, AGE colocalize with activated astrocytes [46]. In 2011, Srikanth et al. showed that the percentage of AGE positive neurons and astroglia increase in Alzheimer with the progression of disease, which might contribute to many aspects of neuronal dysfunction in AD by processes, such as inflammatory activation of microglia, or direct cytotoxicity via formation of free radicals [45], presumably mediated through activation of their receptor RAGE [45]. RAGE binds also the monomeric and fibrillary forms of A*β*. Upon binding of ligands (AGEs and A*β*), RAGE triggers intracellular signaling pathways via phosphatidylinositol-3 kinase, Ki-Ras, and mitogen-activated protein kinases, the Erk1 and Erk2 [17]. Those pathways culminate in the activation of the transcription factor nuclear factor kappa B (NF-*κ*B) and subsequent transcription of a number of genes, including endothelin-1, tissue factor, interleukin (IL)-1, IL-6, and tumor necrosis factor (TNF)-*α* [17, 18, 47]. Activation of NF-*κ*B and induction of cytokines can also contribute to neuronal plasticity and the cellular response to neurodegeneration [48]. RAGE-induced signaling results in an initial neuroprotective effect [27], while it contributes to cellular dysfunction when chronically activated [17]. Notably, NF-*κ*B induces the expression of RAGE, leading to a positive loop, which amplify the cellular response to external stress [17]. Furthermore, the engagement of RAGE by AGEs triggers the generation of ROS via the activation of NADPH oxidase (NOX) [45]. NOX catalyzes the reduction of molecular O_2_ by donating an electron from reduced nicotinamide adenine dinucleotide phosphate to generate superoxide. NOX plays an important role in AD-induced ROS release. Thus, RAGE can be considered a key mediator of age-induced oxidative stress by its capability to amplify a stress signal, contributing to the progression of neurodegenerative processes in sporadic AD.

## 3. Role of Neuronal RAGE in AD

The expression level of RAGE is high in rodent cortical neurons during the neonatal period [49], while its presence strongly decreases during maturity with few cortical neurons showing RAGE staining [50]. However, increased RAGE expression in the brain parallels the progression of neurodegenerative diseases such as AD and Huntington's disease [11, 21, 50, 51]. Notably, AD patients show enhanced RAGE, A*β*, and AGEs expression in the whole hippocampus, especially in dentate gyrus neurons and in CA3 pyramidal neurons, which parallels the impairment of short-term memory that is characteristic of AD due to neuronal dysfunction in the hippocampus [11].

 Chronic activation of RAGE affects neuronal function by activating various signaling pathways, promoting both the phosphorylation of tau and the production of A*β*, as well as it mediates A*β* toxicity.

 A recent study demonstrates that injection of AGEs in the rat hippocampus leads to RAGE-dependent tau hyperphosphorylation, spatial memory deficit, and impaired synaptic transmission as demonstrated by inhibition of long-term potentiation (LTP) in AGEs treated rats [52]. Altered synaptic transmission correlated with RAGE-dependent tau hyperphosphorylation that is due to inhibition of Akt and subsequent activation of GSK3. RAGE activation leads also to alterations of the postsynaptic machinery and decreased density of dendritic spines [52]. Interestingly, AD is also characterized by nonenzymatically glycated tau [53], which induces neuronal oxidative and subsequent release of A*β*, further supporting the role of metabolic dysfunction in sporadic AD.

 RAGE induces the expression of BACE 1, a key enzyme implicated in the production of A*β* after stimulation with either AGEs or A*β*. RAGE triggering leads to NF-*κ*B nuclear translocation, which in turn enhances the expression of RAGE leading to a vicious circle producing RAGE-dependent cellular dysfunction [17, 18, 47]. In the brain of a rat model of diabetes, activation of RAGE with AGEs leads to NF-*κ*B-dependent expression of BACE1 [16]. AGEs are increased in the brain of AD patients [16]. These results confirm the role of AGEs and RAGE as molecules linking DM and AD. Another study demonstrated that RAGE induces BACE1 expression in an AD mice model and in A*β*-stimulated neuronal cells in vitro, by stimulating intracellular calcium and activating nuclear factor of activated T cell 1 (NFAT) [54]. Although the signaling pathway induced by RAGE upon A*β* stimulation differs compared to the study describing the role of AGEs-stimulated RAGE in the DM animal model, both reports underline the role of RAGE in promoting the expression of BACE1, which enhances A*β* production in the brain.

 Several evidences clearly demonstrate that RAGE strongly enhances A*β*-induced neuronal dysfunction in AD transgenic (Tg) mice that overexpress a mutant form of human amyloid precursor protein (mAPP), which enhances the production of A*β*1-42 in neuronal cells. These mice show A*β*-induced synaptotoxicity in the absence of amyloid plaque [55]. Overexpression of RAGE anticipates the onset of neuronal dysfunction in double transgenic mice overexpressing neuronal mAPP and RAGE (Tg mAPP/RAGE) compared to the single Tg expressing mAPP only [56]. RAGE-dependent anticipation of neuronal dysfunction was demonstrated by earlier impairment of learning/memory in double Tgs mAPP/RAGE compared to single Tg mAPP mice. Exacerbation of memory impairment correlates with an anticipation of synaptic dysfunction in the hippocampus of double Tgs as demonstrated by alteration of LTP [56]. A decrement of cholinergic fibers and presynaptic terminals appears earlier in mAPP/RAGE compared to map mice [56]. On the contrary, inhibition of RAGE confers a neuroprotective effect in AD mice, as demonstrated in double Tg mice expressing mAPP and a dominant negative form of RAGE (DNRAGE) in neurons [56]. DNRAGE encodes for a truncated form of RAGE lacking the intracellular domain necessary to induce RAGE-mediated signaling, while maintaining the extracellular domain for ligand binding. DNRAGE expression blocks the function of endogenous RAGE [56]. Double Tg mAPP/DNRAGE performed better in learning and memory test compared to single Tg mAPP. Expression of DNRAGE completely prevented neuropathologic changes such as loss of cholinergic fibers induced by mAPP [56].

 Another area of the brain that is important in memory process and is early affected in AD is the entorhinal cortex. In agreement, oligomeric A*β*1-42 impairs LTP in slides derived from this brain area of wild-type (wt) mice [57]. A*β*-induced LTP alteration is inhibited by coaddition of anti-RAGE IgG. Similarly, A*β* has not any effect on slides derived from RAGE null mice or Tg mice expressing neuronal DNRAGE [57]. Moreover, this study demonstrated that RAGE is implicated in A*β*-induced synaptic dysfunction by activating the pathway of p38 MAPK [57, 58]. RAGE plays a key role also in A*β*-dependent inhibition of synaptic plasticity in intracortical circuits of the visual cortex, and RAGE blockade confers a neuroprotective effect against A*β*-induced neuronal dysfunction [59]. In contrast, in Arc/swe AD mice, which overexpress hAPP carrying the Swedish (swe) mutation, which enhances A*β* production, and the arctic (arc) mutation in A*β* sequence, which leads to a faster aggregation of A*β* [60], the knockout of RAGE has only a minimal effect on A*β* load and does not ameliorate synaptic dysfunction. Taken together, these data underline the differences in the pathologic mechanisms implicated in sporadic and familial AD, supporting the hypothesis that RAGE plays a key function specifically in the progression of sporadic AD.

 Several studies demonstrated that A*β* and AGEs affect energy metabolism by decreasing mitochondrial activity and induce neurodegeneration by producing mitochondrial damage [19, 61]. Injection of A*β*25-35 toxic fragment in rat CA1 hippocampus enhances RAGE expression in CA1, which parallels with a 56% decrement in mitochondrial activity and the presence of neurodegenerative events [62]. RAGE is involved in the uptake of A*β* and A*β* targeting to mitochondria in cortical neurons, leading to a decrement in the activity of a key mitochondrial respiratory enzyme, the cytochrome c oxidase (COX IV) [63]. Blockade of RAGE with anti-RAGE IgG or A*β* treatment of neurons derived from RAGE null mice diminishes A*β* targeting to mitochondria and subsequent mitochondrial dysfunction. Inhibition of RAGE-dependent p38 MAK activation blocks A*β* targeting to mitochondria and the subsequent mitochondrial damage. RAGE colocalizes with A*β* in an intracellular compartment *in vivo* in pyramidal cells of the CA3 region of the hippocampus in the Tg mAPP mice [63] further supporting the role of RAGE in A*β*-mediated neurodegeneration by affecting mitochondrial function. Moreover, these studies demonstrate that RAGE inhibition confers a neuroprotective effect against A*β*-mediated toxicity.

 Several studies demonstrated that RAGE triggering induces neurite outgrowth and neuronal differentiation [35, 64–69]. Furthermore, various studies including our own demonstrate that RAGE is required for the repair of the injured nerve [27, 70, 71]. Thus, RAGE plays a dual function: it can mediate neurite outgrowth and neuronal repair, while it induces neuronal dysfunction when chronically activated. Because of the dual function of RAGE, compounds capable to block the chronic activation of RAGE can exert a neuroprotective effect in AD.

## 4. Role of RAGE in Glial Cells and Inflammation in AD

 Several evidences substantiate the association between neuroinflammatory mechanisms and the pathological events leading to neuronal dysfunction and neurodegeneration. The brain of AD patients shows chronic inflammation that is characterized by the presence of reactive astrocytes and activated microglia [72]. In healthy physiological conditions, astrocytes are necessary to maintain brain homeostasis and neuronal function. They provide metabolic support for neurons in form of lactate, glutamate uptake and its conversion into glutamine, and synthesis of antioxidant enzymes [72]. Microglial cells represent the innate immune system in the brain as they can have a role as cerebral macrophages as well they recruit and stimulate astrocytes [73]. Neuroinflammation and microglial activations regulate the delicate balance of immune response and neuronal homeostasis. The innate immune responses of glial to injurious insults or activating stimuli lead to beneficial outcomes, such as phagocytosis of pathogens, and production of reparative and protective factors. However, chronic activation of glial cells results in overproduction of proinflammatory factors, disturb homeostasis, and ultimately exacerbates neuronal dysfunction enhancing the progression of neuropathology [74]. Activated astrocytes in AD fails in providing metabolic support to neurons, contributing in inducing neurodegeneration [72]. Moreover, the activation of astrocytes and microglia leads to chronic oxidative stress in AD patients, further contributing to neurodegenerative processes [72]. Noteworthy, oxidative stress leads to the formation of AGEs, which will activate RAGE [72]. Several studies including our own demonstrated that activation of RAGE induces oxidative stress and inflammation [18, 26, 27, 47, 75, 76]. Thus, glial inflammation and subsequent AGEs formation in the presence of A*β* lead to a positive feedback loops by which inflammation in AD increases proinflammatory signaling. Inflammation enhances the processing of APP in astrocytes by inducing BACE1 expression, leading to A*β* deposition, further activating RAGE [45]. Moreover, RAGE ligands enhance the expression of RAGE itself, leading of a positive loop that induces the expression of RAGE and subsequent oxidative stress and inflammation, which in turn sustain the formation of AGEs and A*β* [17]. Interaction of A*β* with RAGE results in increased expression of macrophage colony stimulating factor (M-CSF) in neuronal cells [77]. Stimulation of microglia by M-CSF results in enhanced cell survival in cell stress conditions, proliferation and induction of proinflammatory gene expression, which leads to chronic inflammation and contributes to neurodegenerative processes [77]. Indeed, M-CSF induces cell survival in microglial cells, which express c-fms receptor. On the contrary, neuronal cells do not express c-fms receptor and do not benefit of M-CSF prosurvival effects, while they are further affected by the proinflammatory reaction of glial cells [19]. The combination of AGEs and A*β* synergistically induces the expression of proinflammatory cytokines, such as TNF-*α*, IL-6, and M-CSF [45]. Moreover, A*β* induces the expression and secretion of IL-1*β* in glial cells [45] via RAGE [27]. RAGE is overexpressed in the microglial cell in AD patients [78] and in an AD mice model (mAPP Tg) [56]. Activated microglia exacerbate A*β*-induced neuronal toxicity [74], and RAGE is a key mediator of activated microglial effects in AD neuronal dysfunction [78, 79]. Targeted overexpression of RAGE in the microglia of mAPP Tg mice (double Tg mAPP/micRAGE) enhances the expression of proinflammatory cytokines, increases A*β* production, and accelerates neuropathologic changes compared to single Tg mAPP, as demonstrated by anticipation of cholinergic fiber loss and alteration in learning and memory [78]. Conversely, targeted overexpression of a dominant negative form of RAGE in microglia of mAPP Tg mice (double Tg mAPP/micDNRAGE) leads to a decrement of cytokines and A*β* production and ameliorates neuronal dysfunction compared to the single Tg mAPP [78]. In addition, targeted overexpression of a dominant negative form of RAGE in microglia (double Tg mAPP/micDNRAGE) attenuates A*β*-induced synaptic dysfunction and A*β*-dependent inhibition of long-term depression (LTD) in entorhinal cortex [79], demonstrating that RAGE blockade inhibits A*β*-induced neuronal dysfunction.

 In summary, several studies support the hypothesis that RAGE-mediated inflammation in AD contributes in inducing neuronal dysfunction. On the contrary, these studies demonstrate that inhibition of RAGE activation induces neuroprotection and ameliorates AD progression.

## 5. Role of RAGE and Vascular Dysfunction in AD

 The potential link between cerebral blood vessel disease and Alzheimer's is one promising area of research. Vascular disease in the aged appears to have strong implications for neurodegeneration leading to dementia. Preliminary studies indicate that a broad spectrum of cerebrovascular lesions could lead to a decline in cognitive function. Moreover, nearly 80 percent of individuals with AD also have cardiovascular disease at autopsy, supporting the hypothesis that systemic vascular factors are risk factors for developing AD. This risk encompasses different forms of cardiovascular disease, including coronary artery disease, carotid atherosclerosis, history of hypertension or high cholesterol, type II diabetes, and stroke or transient ischemic attacks [3]. Indeed, another hypothesis accounting for the pathogenesis of AD is the impairment of the blood brain barrier (BBB) [23]. Cerebral blood vessels undergo profound changes with aging and in AD [80]. The BBB blocks the free diffusion of circulating molecules, leukocytes, and monocytes into the brain interstitial space. Moreover, the BBB plays a key role in regulating the glial and neuronal environment. The BBB is constituted by endothelial cells fused by high-resistance tight junctions, in order to separate the blood from the brain. The disruption of the tight junctions affects the regulated transport of molecules and monocytes between blood and brain and brain and blood and induces angiogenesis and vessels regression, as well as brain hypoperfusion and inflammation, promoting ultimately synaptic dysfunction and neurodegeneration. Alterations of the BBB, vascular density, fragmentation of vessels, alteration of the basement membranes, and a decrement of mitochondria in the BBB occur in AD [80]. Notably, BBB dysfunction is associated to several risk factors for AD, such as stroke, cerebrovascular ischemia, hypertension, and mutation in the ApoE gene, which represents the only validated genetic risk factor of AD [3]. Since the large majority of AD cases are sporadic, it has been recently hypothesized that the accumulation of A*β* into the brain and around blood vessels is due in an alteration of clearance of A*β* from the brain and an enhanced transport of A*β* into the brain [22]. In agreement, Tg2576 AD mice display enhanced BBB permeability compared to control mice at 4 months of age, before the appearance of plaque deposition and memory impairment [81]. A correlation between BBB dysfunction and AD has been demonstrated in AD patients. Noteworthy, BBB impairment in these patients was not associated with vascular diseases risk for AD, suggesting that the mechanisms inducing BBB alterations in AD differ from that one implicated in vascular dementia [82].

RAGE is upregulated in AD brain vasculature [10, 11, 50]. *In vivo* studies show a RAGE-dependent transport of A*β*1-40 and A*β*1-42 into the hippocampus and cortex, which is inhibited by anti-RAGE blocking antibodies. The transport of A*β* is strongly impaired and undetectable in RAGE null mice [23]. RAGE-mediated transport of A*β* leads to neurovascular stress, induction of the expression of TNF-*α* and IL-6, which are detected mostly at the level of neurons. Notably, infusion with physiological concentration of A*β* (50 pM) does not induce the expression of proinflammatory cytokines, while neurovascular inflammation is detected when pathological concentrations of A*β* (4.5 nM) are infused in the mice [23]. Moreover, A*β*-RAGE interaction on the BBB induces vasoconstriction by promoting the expression of endothelin-1. Notably, infusion of anti-RAGE IgG ameliorates vascular dysfunction and blocks endothelin-1 expression in Tg2576 AD mice [23].

It has been demonstrated that blood or BM-derived monocytes infiltrate the AD brain, enhancing inflammation [83]. Antibodies against RAGE inhibit A*β*-induced monocytes transmigration across the BBB [84], further demonstrating the key role of RAGE in promoting neurovascular inflammation in AD. Thus, RAGE expressed in brain microvessels participates in AD by enhancing A*β*-transport across the BBB and promoting neurovascular inflammation. Conversely, inhibition of RAGE is beneficial by blocking A*β* transport across the BBB and the subsequent inflammatory response.

## 6. RAGE-TXNIP Axis: Evidence of a Novel Pathway Induced by RAGE in AD

**Figure 1:**
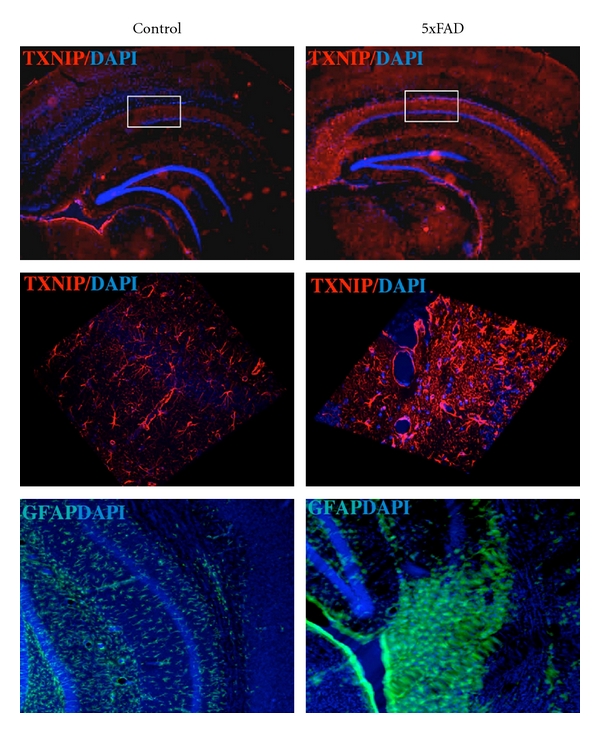
TXNIP is overexpressed in the hippocampus of the 5xFAD mice. Top: Floating brain slices were incubated 24 h with mouse anti-TXNIP monoclonal antibody in PBS 3% BSA, 0.1% Triton X-100 (blocking) at 4°C. Slides were washed 3 times for 15 min with PBS and incubated for 45 min with TRITC-conjugated secondary antibody (red). Nuclei were stained by incubating the slides with Hoecst (blue) together with the secondary antibody. Slides were mounted using mounting medium and analyzed with confocal microscopy (Zeiss). Center: Confocal analysis and 3 dimensional reconstruction (Zen software of Zeiss) of TXNIP staining in the hippocampus. Bottom: Floating brain slices were incubated 2 h at room temperature with rabbit anti-GFAP polyclonal antibody in PBS 3% BSA, 0.1% Triton X-100 (blocking). Slides were washed 3 times for 15 min with PBS and incubated for 45 min with FITC-conjugated secondary antibody (green). Slides were mounted using mounting medium and analyzed with confocal microscopy (Zeiss). These results are representative of 4 independent experiments (4 animals).

**Figure 2:**
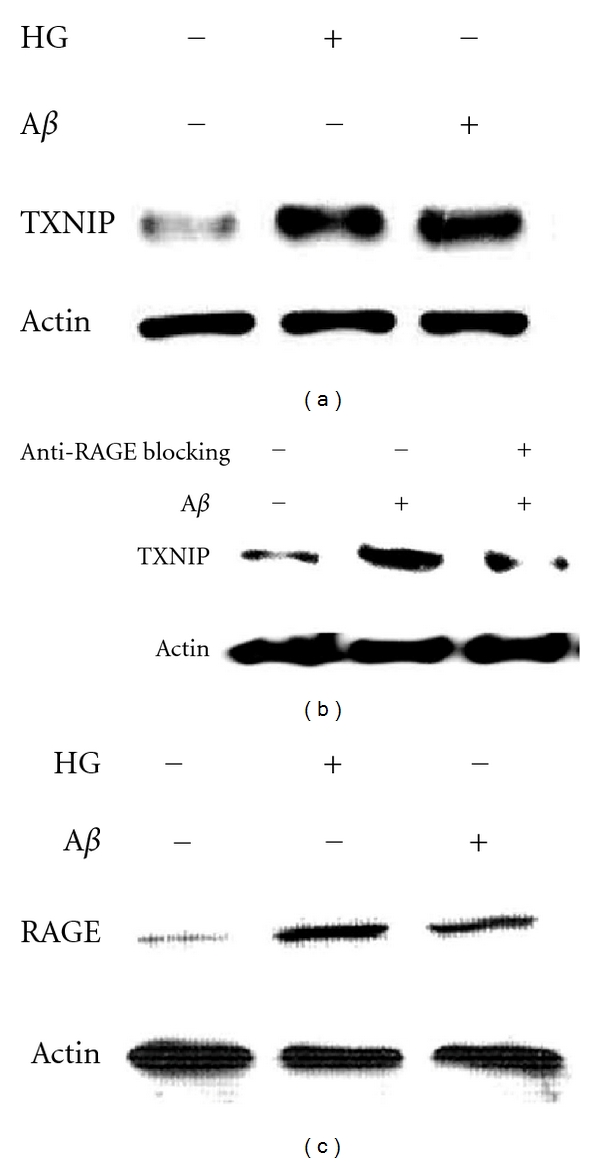
A*β* induces RAGE-dependent TXNIP expression in RBE4 brain endothelial cells. RBE4 cells were maintained 5 days in differentiation medium (F10/MEM, 2.5% FCS, hydrocortisone 14 *μ*M, Hepes 10 mM, bFGF 1 *μ*g/mL). RBE4 cells were stimulated for 6 h with either A*β*1-42 (3 *μ*M) or HG (25 mM) in differentiation medium. Cells were lysed in RIPA buffer. TXNIP expression was analyzed by western blotting using a mouse anti-TXNIP monoclonal antibody (MBL). Protein loading was analyzed by western blotting of actin. (b) RBE4 cells were maintained as described in (a) and stimulated for 6 h with either A*β*1-42 (3 *μ*M) both in the absence or presence of an anti-RAGE blocking antibody (R&D system). TXNIP expression and protein loading were analyzed by western blotting as in (a). (c) RBE4 cells were maintained as described in (a) and stimulated for 6 h with either A*β*1-42 (3 *μ*M) or HG (25 mM) in differentiation medium. RAGE expression was analyzed by western blotting using a rabbit anti-RAGE polyclonal antibody (Santa Cruz). Protein loading was analyzed by western blotting of actin. These data are representative of 3 independent experiments.

**Figure 3:**
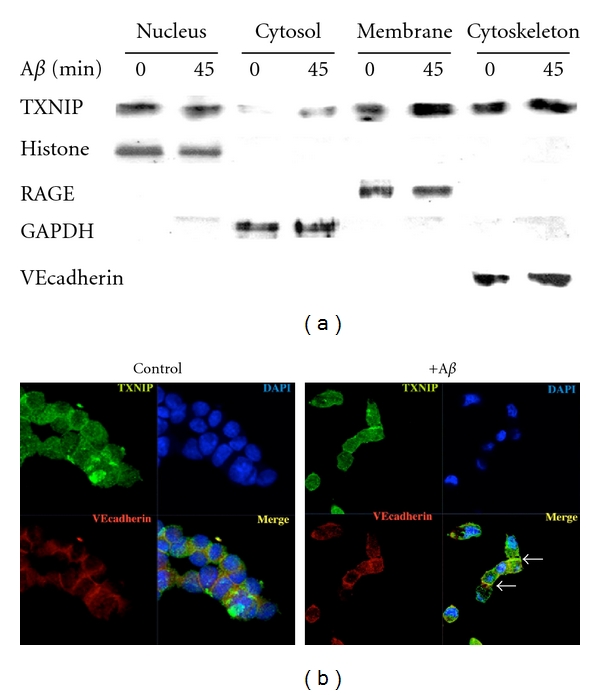
A*β* enhances TXNIP translocation to the plasma membrane. (a) RBE4 cells were maintained 5 days in differentiation medium (F10/MEM, 2.5% FCS, hydrocortisone 14 *μ*M, Hepes 10 mM, bFGF 1 *μ*g/mL). RBE4 cells were stimulated for 45 min with A*β*1-42 (3 *μ*M). Subcellular fractions were obtained using a cell fractionation kit (Biorad) according to the manufacturer instruction. The presence of TXNIP, RAGE, VE-cadherin, and histone H3 were analyzed by western blotting. (b) RBE4 cells were maintained as described in (a) and stimulated for 45 min with A*β*1-42 (3 *μ*M). Cells were fixed in PBS containing 4% PFA and permeabilized 10 min in PBS 0.1% Triton X-100. Cells were maintained 1 h in blocking solution (PBS 3% BSA) at room temperature and then incubated over/night at 4°C with a rabbit anti-TXNIP polyclonal antibody (Invitrogen) and a mouse anti-VEcadherin monoclonal antibody (Santa Cruz biotechnology) in blocking solution. Cells were washed 3 times for 15 min with PBS and incubated with the appropriate secondary antibody. Nuclei were stained with Hoecst. Immunofluorescence was analyzed by a confocal microscopy (Zeiss). These data are representative of 3 independent experiments.

Recent studies using the human brain indicate that insulin signaling is impaired in the AD brain. In neurons, this insulin signaling plays a key role in modulating synaptic function and neuronal senescence [85]. Spatial learning in rats induces the expression of insulin receptor and of insulin receptor substrate 1 (IRS 1) in the hippocampus. Moreover, insulin regulates tau phosphorylation, a hallmark of AD [86]. Insulin also regulates glucose metabolism in the brain by modulating the expression of glucose transporters [85]. TXNIP is an intriguing candidate molecule that may provide a common link between brain insulin resistance and AD. TXNIP was initially characterized for its capability to inhibit thioredoxin, leading to oxidative stress [26, 87]. However, recent studies demonstrated that TXNIP regulates glucose metabolism [88, 89], and its expression is associated to the senescence process [90]. Notably, TXNIP null mice are resistant to diabetes, showing that TXNIP is necessary for the induction of insulin resistance [34]. In the mice brain, TXNIP is expressed in the nuclei of astrocytes and at low level in some neurons. TXNIP expression is low in the hippocampus, while it is expressed constitutively in hypothalamic neurons where it senses nutrients excess [91, 92]. TXNIP is also an early induced gene by apoptosis in cerebellar neurons [93]. Insulin modulates memory by promoting the expression of N-methyl-D-aspartate (NMDA) receptors, which enhances neuronal Ca^2+^ influx, consolidating neuronal synaptic association and promoting LTP [85]. Synaptic activity inhibits TXNIP expression in neurons through NMDA receptor (NMDAR) activation. Blockade of NMDAR enhances TXNIP expression, promoting neuronal vulnerability to oxidative damage [94]. Notably, A*β* affects NMDAR function and trafficking [95], further supporting the hypothesis that TXNIP may be implicated in AD. However, no any study up to now investigated TXNIP expression in AD. For this reason, we analyzed the expression of TXNIP in the brain of the 5xFAD mice model of AD. 5xFAD expresses neuronal human APP carrying three AD familiar mutation (Swedish, Florida, London) and presenilin 1 (PS1) containing 2 mutations (M146L and L286V) [96]. Since TXNIP is implicated in senescence, we used the 5xFAD mice that display an early AD phenotype. Indeed, 5xFAD mice show intraneuronal A*β* accumulation at 2 months age, impaired learning/memory and reduction of synaptophysin levels at 4 months age, and cortical neuronal apoptosis at 9 months age [96]. TXNIP was overexpressed in the hippocampus (Figure 1 top and middle) and in the entherinal cortex (*not shown*) of 5xFAD mice at 6 months of age compared to control mice. To investigate TXNIP expression, we used a mouse anti-TXNIP monoclonal antibody (clone JY2, MBL). Similar results were obtained using a rabbit anti-TXNIP polyclonal antibody (Invitrogen). TXNIP overexpression paralleled enhanced astrogliosis, as demonstrated by increased expression of glial fibrillary acidic protein in the hippocampus (Figure 1 bottom). The expression of TXNIP in 5xFAD brain capillary endothelial cells in the hippocampus was detected using both monoclonal and the polyclonal anti-TXNIP antibodies (*not shown*). Noteworthy, hippocampus and entorhinal cortex are associated to the early learning/memory impairment in AD. Since we previously demonstrated that RAGE induces TXNIP expression in retinal endothelial cells leading to chronic inflammation and ultimately inducing neurodegeneration in diabetic retina [26, 30], we studied whether A*β* induces TXNIP expression in brain derived endothelial cells (RBE4). RBE4 cells were maintained in differentiation medium (F10/MEM, 2.5% FCS, hydrocortisone 14 *μ*M, Hepes 10 mM, bFGF 1 *μ*g/mL) [97] for 5 days, before stimulated for 6 h with A*β*b1-42 (3 *μ*M). Since hyperglycemia (HG) induces TXNIP expression [26, 87], as control we stimulated RBE4 cells for 6 h with HG (25 mM glucose). Both HG and A*β* induced TXNIP expression in RBE4 cells (Figure 2(a)). A*β*-induced TXNIP expression was RAGE-dependent, because an anti-RAGE blocking antibody (R&D system) [98] completely inhibited A*β*-induced TXNIP expression in RBE4 cells (Figure 2(b)). Moreover, RBE4 cells treated for 6 h with either HG (25 mM) or A*β* (3 *μ*M) displayed enhanced RAGE expression compared to control cells (Figure 2(c)). It has been recently shown that TXNIP translocation to the plasma membrane in endothelial cells participates in cell migration leading to angiogenesis [99]. Since angiogenesis occurs in AD [80], we investigated whether A*β* treatment induces TXNIP translocation in RBE4 cells. Fractionation analysis of cell extracts reveals that 45 min of A*β* treatment increases the cofractionation of TXNIP with the plasma membrane marker VE-cadherin (Figure 3(a)). This result was confirmed by immunofluorescence analysis of TXNIP subcellular localization in the absence or presence of A*β* treatment, which displays an enhanced colocalization of TXNIP with VE-cadherin following A*β* treatment (Figure 3(b)). We also observed an enhanced cofractionation of TXNIP with the cytoskeletal fraction following A*β* treatment (Figure 3(a)), which is confirmed by immunofluorescence analysis showing enhanced colocalization of TXNIP with actin following A*β* treatment (data not shown). Notably, it has been recently demonstrated that triggering of RAGE in endothelial cells leads to altered actin reorganization and membrane resealing, participating in vascular dysfunction [100].

These data strongly imply that RAGE-TXNIP axis contributes to vascular dysfunction in AD, suggesting that RAGE-TXNIP axis is a novel therapeutic target to ameliorate AD.

## 7. Pharmacological Treatment to Ameliorate AD Progression by Blocking RAGE

Since RAGE is implicated in AD progression by orchestrating cellular dysfunction in various cell types, a pharmacological treatment aimed to inhibit RAGE chronic activation would be beneficial in ameliorating AD. The small molecule PF-04494700 inhibits RAGE by blocking the interaction of the receptor with its ligands such as A*β*, AGEs, HMGB1, and members of the proinflammatory S100 family members [101]. Thus, PF-04494700 was thought to be capable to ameliorate AD by inhibiting both inflammation and A*β*-induced neurodegeneration. An initial 10-week-long phase 2 safety trial demonstrated a good safety profile of PF-04494700 in AD patients, even if there was not significant clinical amelioration during the short observation period [101]. Thus, a long-term clinical trail was initiated with three group of treatment: one group received placebo, the second 20 mg/day of PF-04494700, and the third 5 mg/day of the drug, and the researcher analyzed Alzheimer's Disease Assessment-cognitive subscale (ADAS-cog) score, safety indicators, additional cognitive tests, structural magnetic resonance imaging (MRI) measurements, A*β* imaging by positron emission tomography (PET), and levels of the biomarkers A*β* and tau in cerebrospinal fluid (CSF). However, the trial was discontinued after 12 months because the highest dose of PF-04494700 resulted in worsening the ADAS-cog score and side effects, while the lower dose was safe (see Alzheimer Research Forum article: “Door Slams on RAGE” 9th November 2011 http://www.alzforum.org/new/detail.asp?id=2960). Therefore, the use of this drug to ameliorate AD is still debatable. Although the clinical trial was abandoned, the researchers continued to follow the patients and they collected data obtained from visiting these patients after 18 months from the start of the trial. When Douglas Galasko presented the completed data set during the 4th International Conference on Clinical Trials on Alzheimer's Disease (CTAD; November 3–5, 2011, in San Diego, CA, USA), he notably showed that patient, who had received the low dose of PF-04494700 showed an improved ADAS-cog score after 18 months, when compared to the placebo group, even if they were taken off the treatment with PF-04494700 after 12 months. Thus, Galasko suggests that it was an error to stop the clinical trial, at least with the low-dose group. The researcher also reported that the high-dose group completely recovered with the ADAS-cog score after 18 month; thus, the toxic effect was reversible. He did not explain the reason of the toxicity induced by the higher dose of PF-04494700. As outlined in the present, RAGE participates in neurite outgrowth, and RAGE is highly expressed in brain neurons during the development. The higher dose of PF-04494700 might thus block or at least interfere with the yet mot clearly defined physiological functions of RAGE, thereby affecting neurogenesis. On the contrary, the lower dose of PF-04494700, which was beneficial in the long time, suggests that the inhibition of chronic RAGE activation can ameliorate AD progression and imply follow-up studies using low dose of PF-04494700 to inhibit RAGE-induced chronic neurovascular dysfunction.

## 8. Conclusions and Hypothesis

**Figure 4:**
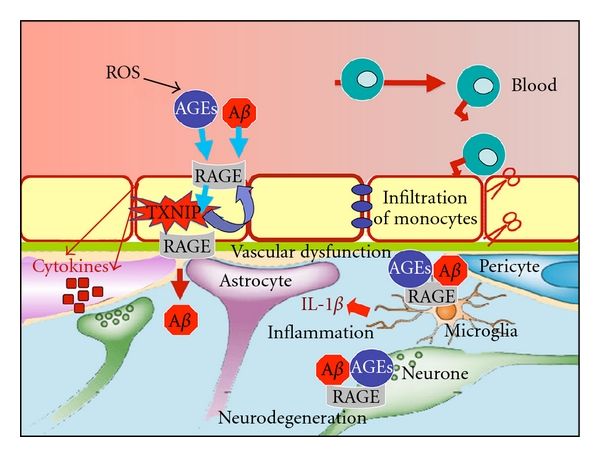
PF-04494700, an inhibitor of RAGE, can ameliorate sporadic AD and promote neuroprotection by blocking RAGE activation in various cell type. Aging-induced oxidative stress leads to the formation of AGEs, which activate RAGE together with A*β* in various cell type. Triggering of RAGE at the BBB leads to TXNIP expression and subsequent inflammation, BBB leakage, and monocytes infiltration. Moreover, RAGE triggering induces a positive feedback loop enhancing RAGE expression, resulting in enhanced transport of A*β* from the blood to the brain. RAGE activation in glial cells promotes proinflammatory gene expression, which enhanced A*β* production inside the brain and neurotoxicity. RAGE triggering in neuronal cells induces oxidative stress and the production of M-CSF, leading to inflammation. Thus, activation of RAGE in different cell types orchestrates the neuroinflammatory processes that ultimately lead to neurodegeneration. Thus, treatments aimed to inhibit chronic RAGE activation will confer a neuroprotective effect by blocking RAGE-mediated neurovascular dysfunction.

Herein, we summarize all studies indicating that RAGE participates in sporadic AD progression by activating several pathways in different cell types, particularly BBB, glia, and neurons (Figure 4). These pathways converge and ultimately lead to synaptic dysfunction and neurodegeneration. We also report ongoing studies demonstrating that RAGE participates in AD progression by inducing TXNIP expression. We previously demonstrated that RAGE-TXNIP axis is induced in different cell types and promotes inflammation [26, 27]. Moreover, we have shown that enhanced TXNIP expression in diabetes ultimately leads to neurodegeneration [30]. In the present paper, we show that RAGE-TXNIP axis is induced in brain endothelial cells. In addition, we demonstrate for the first time that TXNIP is early overexpressed in the hippocampus of an AD mouse model. Several studies suggest that brain insulin resistance is implicated in AD progression. However, the molecular mechanisms leading to brain insulin resistance in AD are still unknown. Our data are suggesting that RAGE may induce brain insulin resistance by enhancing TXNIP expression. Only one study demonstrated that RAGE triggering induces insulin resistance and impairs glucose uptake in skeletal muscle [102]. Induction of RAGE-TXNIP axis in AD brain can further demonstrate the role of RAGE in amplifying age-induced oxidative stress. Indeed, TXNIP induces oxidative stress. The analysis of A*β*-induced TXNIP expression in glial and neuronal cells is under investigation. However, we and other demonstrated that TXNIP is necessary to induce IL-1*β* expression [27, 103] and to promote neurodegeneration [30, 93]. Thus, we hypothesize that RAGE-TXNIP axis participates in AD progression by activating a concerted action of oxidative stress, inflammation, vascular dysfunction, and neurodegeneration.

We also hypothesize that pharmacological treatments aimed to inhibit chronic RAGE activation will be beneficial in blocking neurovascular dysfunction in AD, thereby conferring a neuroprotective effect by restoring the physiological function of RAGE and TXNIP that are implicated in neuronal differentiation and repair. Thus, a prolonged treatment with a low dose of PF-04494700 might block the effects induced by RAGE chronic activation and ameliorate AD progression.

## References

[1] Hardy J, Selkoe DJ (2002). The amyloid hypothesis of Alzheimer’s disease: progress and problems on the road to therapeutics. *Science*.

[2] Kern A, Behl C (2009). The unsolved relationship of brain aging and late-onset Alzheimer disease. *Biochimica et Biophysica Acta*.

[3] Qiu C, Kivipelto M, Von Strauss E (2009). Epidemiology of Alzheimer’s disease: occurrence, determinants, and strategies toward intervention. *Dialogues in Clinical Neuroscience*.

[4] Recuero M, Vicente MC, Martínez-García A (2009). A free radical-generating system induces the cholesterol biosynthesis pathway: a role in Alzheimer’s disease. *Aging Cell*.

[5] Faller P (2009). Copper and zinc binding to amyloid-*β*: coordination, dynamics, aggregation, reactivity and metal-ion transfer. *ChemBioChem*.

[6] Perrone L, Mothes E, Vignes M (2010). Copper transfer from Cu-A*β* to human serum albumin inhibits aggregation, radical production and reduces A*β* toxicity. *ChemBioChem*.

[7] Lesné S, Kotilinek L, Ashe KH (2008). Plaque-bearing mice with reduced levels of oligomeric amyloid-*β* assemblies have intact memory function. *Neuroscience*.

[8] Selkoe DJ (2008). Soluble oligomers of the amyloid *β*-protein impair synaptic plasticity and behavior. *Behavioural Brain Research*.

[9] Bierhaus A, Nawroth PP (2009). The Alzheimer’s disease-diabetes angle: inevitable fate of aging or metabolic imbalance limiting successful aging. *Journal of Alzheimer’s Disease*.

[10] Takeda S, Sato N, Uchio-Yamada K (2010). Diabetes-accelerated memory dysfunction via cerebrovascular inflammation and A*β* deposition in an Alzheimer mouse model with diabetes. *Proceedings of the National Academy of Sciences of the United States of America*.

[11] Valente T, Gella A, Fernàndez-Busquets X, Unzeta M, Durany N (2010). Immunohistochemical analysis of human brain suggests pathological synergism of Alzheimer’s disease and diabetes mellitus. *Neurobiology of Disease*.

[12] Bierhaus A, Hofmann MA, Ziegler R, Nawroth PP (1998). AGEs and their interaction with AGE-receptors in vascular disease and diabetes mellitus. I. The AGE concept. *Cardiovascular Research*.

[13] Rahmadi A, Steiner N, Münch G (2011). Advanced glycation endproducts as gerontotoxins and biomarkers for carbonyl-based degenerative processes in Alzheimer's disease. *Clinical Chemistry and Laboratory Medicine*.

[14] Loske C, Gerdemann A, Schepl W (2000). Transition metal-mediated glycoxidation accelerates cross-linking of *β*- amyloid peptide. *European Journal of Biochemistry*.

[15] Vitek MP, Bhattacharya K, Glendening JM (1994). Advanced glycation end products contribute to amyloidosis in Alzheimer disease. *Proceedings of the National Academy of Sciences of the United States of America*.

[16] Guglielmotto M, Aragno M, Tamagno E (2012). AGEs/RAGE complex upregulates BACE1 via NF-*κ*B pathway activation. *Neurobiology of Aging*.

[17] Bierhaus A, Humpert PM, Morcos M (2005). Understanding RAGE, the receptor for advanced glycation end products. *Journal of Molecular Medicine*.

[18] Bierhaus A, Nawroth PP (2009). Multiple levels of regulation determine the role of the receptor for AGE (RAGE) as common soil in inflammation, immune responses and diabetes mellitus and its complications. *Diabetologia*.

[19] Yan SD, Roher A, Chaney M, Zlokovic B, Schmidt AM, Stern D (2000). Cellular cofactors potentiating induction of stress and cytotoxicity by amyloid *β*-peptide. *Biochimica et Biophysica Acta*.

[20] Schmidt AM, Sahagan B, Nelson RB, Selmer J, Rothlein R, Bell JM (2009). The role of RAGE in amyloid-*β* peptide-mediated pathology in Alzheimer’s disease. *Current Opinion in Investigational Drugs*.

[21] Shi DY, Bierhaus A, Nawroth PP, Stern DM (2009). RAGE and Alzheimer’s disease: a progression factor for amyloid-*β*- induced cellular perturbation?. *Journal of Alzheimer’s Disease*.

[22] Deane R, Bell RD, Sagare A, Zlokovic BV (2009). Clearance of amyloid-*β* peptide across the blood-brain barrier: implication for therapies in Alzheimer’s disease. *CNS and Neurological Disorders—Drug Targets*.

[23] Deane R, Yan SD, Submamaryan RK (2003). RAGE mediates amyloid-*β* peptide transport across the blood-brain barrier and accumulation in brain. *Nature Medicine*.

[24] Deane R, Zlokovic BV (2007). Role of the blood-brain barrier in the pathogenesis of Alzheimer’s disease. *Current Alzheimer Research*.

[25] Daborg J, Von Otter M, Sjölander A (2010). Association of the RAGE G82S polymorphism with Alzheimer’s disease. *Journal of Neural Transmission*.

[26] Perrone L, Devi TS, Hosoya KI, Terasaki T, Singh LP (2009). Thioredoxin interacting protein (TXNIP) induces inflammation through chromatin modification in retinal capillary endothelial cells under diabetic conditions. *Journal of Cellular Physiology*.

[27] Sbai O, Devi TS, Melone MAB (2010). RAGE-TXNIP axis is required for S100B-promoted Schwann cell migration, fibronectin expression and cytokine secretion. *Journal of Cell Science*.

[28] Kim SY, Suh HW, Chung JW, Yoon SR, Choi I (2007). Diverse functions of VDUP1 in cell proliferation, differentiation, and diseases. *Cellular & molecular immunology*.

[29] Marcon G, Tell G, Perrone L (2009). APE1/Ref-1 in Alzheimer’s disease: an immunohistochemical study. *Neuroscience Letters*.

[30] Perrone L, Devi TS, Hosoya KI, Terasaki T, Singh LP (2010). Thioredoxin interacting protein (TXNIP) induces inflammation through chromatin modification in retinal capillary endothelial cells under diabetic conditions. *Journal of Cellular Physiology*.

[31] Yamawaki H, Pan S, Lee RT, Berk BC (2005). Fluid shear stress inhibits vascular inflammation by decreasing thioredoxin-interacting protein in endothelial cells. *Journal of Clinical Investigation*.

[32] Aon-Bertolino ML, Romero JI, Galeano P (2011). Thioredoxin and glutaredoxin system proteins-immunolocalization in the rat central nervous system. *Biochimica et Biophysica Acta*.

[33] Craft S (2007). Insulin resistance and Alzheimer’s disease pathogenesis: potential mechanisms and implications for treatment. *Current Alzheimer Research*.

[34] Chutkow WA, Birkenfeld AL, Brown JD (2010). Deletion of the *α*-arrestin protein Txnip in mice promotes adiposity and adipogenesis while preserving insulin sensitivity. *Diabetes*.

[35] Wang L, Li S, Jungalwala FB (2008). Receptor for advanced glycation end products (RAGE) mediates neuronal differentiation and neurite outgrowth. *Journal of Neuroscience Research*.

[36] Harman D (1981). The aging process. *Proceedings of the National Academy of Sciences of the United States of America*.

[37] Beckman KB, Ames BN (1998). The free radical theory of aging matures. *Physiological Reviews*.

[38] Fleming TH, Humpert PM, Nawroth PP, Bierhaus A (2010). Reactive metabolites and AGE/RAGE-mediated cellular dysfunction affect the aging process—a mini-review. *Gerontology*.

[39] Butterfield DA, Sultana R (2007). Redox proteomics identification of oxidatively modified brain proteins in Alzheimer’s disease and mild cognitive impairment: insights into the progression of this dementing disorder. *Journal of Alzheimer’s Disease*.

[40] Smith CD, Carney JM, Tatsumo T, Stadtman ER, Floyd RA, Markesbery WR (1992). Protein oxidation in aging brain. *Annals of the New York Academy of Sciences*.

[41] Lovell MA, Xie C, Markesbery WR (2001). Acrolein is increased in Alzheimer’s disease brain and is toxic to primary hippocampal cultures. *Neurobiology of Aging*.

[42] Markesbery WR, Lovell MA (1998). Four-hydroxynonenal, a product of lipid peroxidation, is increased in the brain in Alzheimer’s disease. *Neurobiology of Aging*.

[43] Bierhaus A, Nawroth PP (2005). Posttranslational modification of lipoproteins—a fatal attraction in metabolic disease? Commentary on: Hone et al., Alzheimer’s disease amyloid-beta peptide modulates apolipoprotein E isoform specific receptor binding. *Journal of Alzheimer’s Disease*.

[44] Thornalley PJ (2003). Protecting the genome: defence against nucleotide glycation and emerging role of glyoxalase I overexpression in multidrug resistance in cancer chemotherapy. *Biochemical Society Transactions*.

[45] Srikanth V, Maczurek A, Phan T (2011). Advanced glycation endproducts and their receptor RAGE in Alzheimer's disease. *Neurobiology of Aging*.

[46] Horie K, Miyata T, Yasuda T (1997). Immunohistochemical localization of advanced glycation end products, pentosidine, and carboxymethyllysine in lipofuscin pigments of Alzheimer’s disease and aged neurons. *Biochemical and Biophysical Research Communications*.

[47] Bierhaus A, Schiekofer S, Schwaninger M (2001). Diabetes-associated sustained activation of the transcription factor nuclear factor-*κ*B. *Diabetes*.

[48] Mattson MP, Camandola S (2001). NF-*κ*B in neuronal plasticity and neurodegenerative disorders. *Journal of Clinical Investigation*.

[49] Hori O, Brett J, Slattery T (1995). The receptor for advanced glycation end products (RAGE) is a cellular binding site for amphoterin. Mediation of neurite outgrowth and co-expression of RAGE and amphoterin in the developing nervous system. *Journal of Biological Chemistry*.

[50] Yan SD, Chen X, Fu J (1996). RAGE and amyloid-*β* peptide neurotoxicity in Alzheimer’s disease. *Nature*.

[51] Anzilotti S, Giampà C, Laurenti D (2012). Immunohistochemical localization of receptor for advanced glycation end (RAGE) products in the R6/2 mouse model of Huntington's disease. *Brain Research Bulletin*.

[52] Li X-H, Lv B-L, Xie J-Z, Liu J, Zhou X-W, Wang J-Z AGEs induce Alzheimer-like tau pathology and memory deficit via RAGE-mediated GSK-3 activation.

[53] Shi Du Yan, Shi Fang Yan, Chen X (1995). Non-enzymatically glycated tau in Alzheimer’s disease induces neuronal oxidant stress resulting in cytokine gene expression and release of amyloid *β*-peptide. *Nature Medicine*.

[54] Cho HJ, Son SM, Jin SM (2009). RAGE regulates BACE1 and A*β* generation via NFAT1 activation in Alzheimer’s disease animal model. *FASEB Journal*.

[55] Mucke L, Masliah E, Yu GQ (2000). High-level neuronal expression of A*β*(1–42) in wild-type human amyloid protein precursor transgenic mice: synaptotoxicity without plaque formation. *Journal of Neuroscience*.

[56] Arancio O, Zhang HP, Chen X (2004). RAGE potentiates A*β*-induced perturbation of neuronal function in transgenic mice. *EMBO Journal*.

[57] Origlia N, Righi M, Capsoni S (2008). Receptor for advanced glycation end product-dependent activation of p38 mitogen-activated protein kinase contributes to amyloid-*β*-mediated cortical synaptic dysfunction. *Journal of Neuroscience*.

[58] Origlia N, Arancio O, Domenici L, Yan SS (2009). MAPK, *β*-amyloid and synaptic dysfunction: the role of RAGE. *Expert Review of Neurotherapeutics*.

[59] Origlia N, Capsoni S, Cattaneo A (2009). A*β*-dependent inhibition of LTP in different intracortical circuits of the visual cortex: the role of RAGE. *Journal of Alzheimer’s Disease*.

[60] Vodopivec I, Galichet A, Knobloch M, Bierhaus A, Heizmann CW, Nitsch RM (2009). RAGE does not affect amyloid pathology in transgenic arcA*β* mice. *Neurodegenerative Diseases*.

[61] Kuhla B, Loske C, Garcia De Arriba S, Schinzel R, Huber J, Münch G (2004). Differential effects of "Advanced glycation endproducts" and *β*-amyloid peptide on glucose utilization and ATP levels in the neuronal cell line SH-SY5Y. *Journal of Neural Transmission*.

[62] Cuevas E, Lantz SM, Tobon-Velasco C (2011). On the in vivo early toxic properties of A*β*
_25–35_ peptide in the rat hippocampus: involvement of the receptor-for-advanced glycation-end-products and changes in gene expression. *Neurotoxicology and Teratology*.

[63] Takuma K, Fang F, Zhang W (2009). RAGE-mediated signaling contributes to intraneuronal transport of amyloid-*β* and neuronal dysfunction. *Proceedings of the National Academy of Sciences of the United States of America*.

[64] Chou DKH, Zhang J, Smith FI, McCaffery P, Jungalwala FB (2004). Developmental expression of receptor for advanced glycation end products (RAGE), amphoterin and sulfoglucuronyl (HNK-1) carbohydrate in mouse cerebellum and their role in neurite outgrowth and cell migration. *Journal of Neurochemistry*.

[65] Huttunen HJ, Fages C, Rauvala H (1999). Receptor for advanced glycation end products (RAGE)-mediated neurite outgrowth and activation of NF-*κ*B require the cytoplasmic domain of the receptor but different downstream signaling pathways. *Journal of Biological Chemistry*.

[66] Huttunen HJ, Kuja-Panula J, Rauvala H (2002). Receptor for advanced glycation end products (RAGE) signaling induces CREB-dependent chromogranin expression during neuronal differentiation. *Journal of Biological Chemistry*.

[67] Huttunen HJ, Kuja-Panula J, Sorci G, Agneletti AL, Donato R, Rauvala H (2000). Coregulation of neurite outgrowth and cell survival by amphoterin and S100 proteins through receptor for advanced glycation end products (RAGE) activation. *Journal of Biological Chemistry*.

[68] Sajithlal G, Huttunen H, Rauvala H, Münch G (2002). Receptor for advanced glycation end products plays a more important role in cellular survival than in neurite outgrowth during retinoic acid-induced differentiation of neuroblastoma cells. *Journal of Biological Chemistry*.

[69] Srikrishna G, Huttunen HJ, Johansson L (2002). N-glycans on the receptor for advanced glycation end products influence amphoterin binding and neurite outgrowth. *Journal of Neurochemistry*.

[70] Ling LR, Trojaborg W, Qu W (2004). Antagonism of RAGE suppresses peripheral nerve regeneration. *FASEB Journal*.

[71] Rong LL, Yan SF, Wendt T (2004). RAGE modulates peripheral nerve regeneration via recruitment of both inflammatory and axonal outgrowth pathways. *FASEB Journal*.

[72] Fuller S, Steele M, Münch G (2010). Activated astroglia during chronic inflammation in Alzheimer’s disease-do they neglect their neurosupportive roles?. *Mutation Research*.

[73] Blasko I, Stampfer-Kountchev M, Robatscher P, Veerhuis R, Eikelenboom P, Grubeck-Loebenstein B (2004). How chronic inflammation can affect the brain and support the development of Alzheimer’s disease in old age: the role of microglia and astrocytes. *Aging Cell*.

[74] Weninger SC, Yankner BA (2001). Inflammation and Alzheimer disease: the good, the bad, and the ugly. *Nature Medicine*.

[75] Bierhaus A, Stern DM, Nawroth PP (2008). RAGE in inflammation: a new therapeutic target?. *Current Opinion in Investigational Drugs*.

[76] Mohamed AK, Bierhaus A, Schiekofer S, Tritschler H, Ziegler R, Nawroth PP (1999). The role of oxidative stress and NF-*κ*B activation in late diabetic complications. *BioFactors*.

[77] Yan SD, Zhu H, Fu J (1997). Amyloid-*β* peptide-receptor for advanced glycation endproduct interaction elicits neuronal expression of macrophage-colony stimulating factor: a proinflammatory pathway in Alzheimer disease. *Proceedings of the National Academy of Sciences of the United States of America*.

[78] Fang, Lue LF, Yan S (2010). RAGE-dependent signaling in microglia contributes to neuroinflammation, A*β* accumulation, and impaired learning/memory in a mouse model of Alzheimer’s disease. *FASEB Journal*.

[79] Origlia N, Bonadonna C, Rosellini A (2010). Microglial receptor for advanced glycation end product-dependent signal pathway drives *β*-amyloid-induced synaptic depression and long-term depression impairment in entorhinal cortex. *Journal of Neuroscience*.

[80] Desai BS, Schneider JA, Li JL, Carvey PM, Hendey B (2009). Evidence of angiogenic vessels in Alzheimer’s disease. *Journal of Neural Transmission*.

[81] Ujiie M, Dickstein DL, Carlow DA, Jefferies WA (2003). Blood-brain barrier permeability precedes senile plaque formation in an Alzheimer disease model. *Microcirculation*.

[82] Farrall AJ, Wardlaw JM (2009). Blood-brain barrier: ageing and microvascular disease—systematic review and meta-analysis. *Neurobiology of Aging*.

[83] Malm T, Koistinaho M, Muona A, Magga J, Koistinaho J (2010). The role and therapeutic potential of monocytic cells in Alzheimer’s disease. *GLIA*.

[84] Giri R, Selvaraj S, Miller CA (2002). Effect of endothelial cell polarity on *β*-amyloid-induced migration of monocytes across normal and AD endothelium. *American Journal of Physiology*.

[85] Watson GS, Craft S (2006). Insulin resistance, inflammation, and cognition in Alzheimer’s Disease: lessons for multiple sclerosis. *Journal of the Neurological Sciences*.

[86] Ke YD, Delerue F, Gladbach A, Götz J, Ittner LM (2009). Experimental diabetes mellitus exacerbates Tau pathology in a transgenic mouse model of Alzheimer’s disease. *PLoS ONE*.

[87] Turturro F, Friday E, Welbourne T (2007). Hyperglycemia regulates thioredoxin-ROS activity through induction of thioredoxin-interacting protein (TXNIP) in metastatic breast cancer-derived cells MDA-MB-231. *BMC Cancer*.

[88] Muoio DM (2007). TXNIP links redox circuitry to glucose control. *Cell Metabolism*.

[89] Parikh H, Carlsson E, Chutkow WA (2007). TXNIP regulates peripheral glucose metabolism in humans. *PLoS Medicine*.

[90] Mousa SA, Gallati C, Simone T (2009). Dual targeting of the antagonistic pathways mediated by Sirt1 and TXNIP as a putative approach to enhance the efficacy of anti-aging interventions. *Aging*.

[91] Blouet C, Schwartz GJ (2011). Nutrient-sensing hypothalamic TXNIP links nutrient excess to energy imbalance in mice. *Journal of Neuroscience*.

[92] Levendusky MC, Basle J, Chang S, Mandalaywala NV, Voigt JM, Dearborn RE (2009). Expression and regulation of vitamin D3 upregulated protein 1 (VDUP1) is conserved in mammalian and insect brain. *Journal of Comparative Neurology*.

[93] Saitoh T, Tanaka S, Koike T (2001). Rapid induction and Ca2+ influx-mediated suppression of vitamin D3 up-regulated protein 1 (VDUP1) mRNA in cerebellar granule neurons undergoing apoptosis. *Journal of Neurochemistry*.

[94] Papadia S, Soriano FX, Léveillé F (2008). Synaptic NMDA receptor activity boosts intrinsic antioxidant defenses. *Nature Neuroscience*.

[95] Decker H, Jürgensen S, Adrover MF (2010). N-Methyl-d-aspartate receptors are required for synaptic targeting of Alzheimer’s toxic amyloid-*β* peptide oligomers. *Journal of Neurochemistry*.

[96] Oakley H, Cole SL, Logan S (2006). Intraneuronal *β*-amyloid aggregates, neurodegeneration, and neuron loss in transgenic mice with five familial Alzheimer’s disease mutations: potential factors in amyloid plaque formation. *Journal of Neuroscience*.

[97] Faria A, Pestana D, Teixeira D (2010). Flavonoid transport across RBE4 cells: a blood-brain barrier model. *Cellular and Molecular Biology Letters*.

[98] Perrone L, Peluso G, Melone MAB (2008). RAGE recycles at the plasma membrane in S100B secretory vesicles and promotes Schwann cells morphological changes. *Journal of Cellular Physiology*.

[99] World C, Spindel ON, Berk BC (2011). Thioredoxin-interacting protein mediates TRX1 translocation to the plasma membrane in response to tumor necrosis factor-*α*: a key mechanism for vascular endothelial growth factor receptor-2 transactivation by reactive oxygen species. *Arteriosclerosis, Thrombosis, and Vascular Biology*.

[100] Xiong F, Leonov S, Howard AC (2011). Receptor for Advanced Glycation End products (RAGE) prevents endothelial cell membrane resealing and regulates F-actin remodeling in a *β*-catenin-dependent manner. *Journal of Biological Chemistry*.

[101] Sabbagh MN, Agro A, Bell J, Aisen PS, Schweizer E, Galasko D (2011). PF-04494700, an oral inhibitor of receptor for advanced glycation end products (RAGE), in Alzheimer disease. *Alzheimer Disease &amp; Associated Disorders*.

[102] Cassese A, Esposito I, Fiory F (2008). In skeletal muscle advanced glycation end products (AGEs) inhibit insulin action and induce the formation of multimolecular complexes including the receptor for AGEs. *Journal of Biological Chemistry*.

[103] Koenen TB, Stienstra R, Van Tits LJ (2011). Hyperglycemia activates caspase-1 and TXNIP-mediated IL-1*β* transcription in human adipose tissue. *Diabetes*.

